# Antimicrobial stewardship program for gastrointestinal surgeries at a Vietnamese tertiary hospital

**DOI:** 10.3389/fmed.2024.1345698

**Published:** 2024-04-17

**Authors:** Hong Tham Pham, Tuong-Anh Mai-Phan, Anh Dung Nguyen, Van-Quang-Huy Nguyen, Minh-Hoang Tran

**Affiliations:** ^1^Faculty of Pharmacy, Nguyen Tat Thanh University, Ho Chi Minh City, Vietnam; ^2^Department of Pharmacy, Nhan Dan Gia Dinh Hospital, Ho Chi Minh City, Vietnam; ^3^Department of Surgical Gastroenterology, Nhan Dan Gia Dinh Hospital, Ho Chi Minh City, Vietnam; ^4^University of Science, VNU-HCM, Ho Chi Minh City, Vietnam; ^5^NTT Hi-Tech Institute, Nguyen Tat Thanh University, Ho Chi Minh City, Vietnam

**Keywords:** antimicrobial stewardship, antimicrobial prophylaxis, gastrointestinal surgery, surgical site infection, Vietnam

## Abstract

**Background:**

Antimicrobial Stewardship Programs (ASP) have been applied widely in high-resource countries to prevent surgical site infections (SSI). Evidence favoring ASP interventions (ASPi) in gastrointestinal surgeries from low and middle-income countries has been limited, especially in antimicrobial prophylaxis. We aimed to investigate this gap at a Vietnamese tertiary hospital.

**Methods:**

We conducted a retrospective cohort study on patients undergoing clean-contaminated surgeries in 2015 who received standard of care (SoC) or SoC + ASPi. Primary outcome was 30-day SSI incidence. Secondary outcomes included length of stay (LoS) after surgery (days), cost of antibiotics, and cost of treatment (USD). Results were controlled for multiplicity and reported with treatment effect and 95% confidence interval (95%CI). A predictive model was built and cross-validated to detect patients at high risk of SSI.

**Results:**

We included 395 patients for analysis (48.1% being female, mean age 49.4 years). Compared to patients receiving SoC, those with SoC + ASPi had a lower incidence of 30-day SSI (−8.8, 95%CI: −16.0 to −1.6, *p* = 0.042), shorter LoS after surgery (−1.1 days, 95%CI: −1.8 to −0.4, *p* = 0.004), and lower cost of antibiotics (−37.3 USD, 95%CI: −59.8 to −14.8, *p* = 0.012) and treatment (−191.1 USD, 95%CI: −348.4 to −33.8, *p* = 0.042). We estimated that by detecting patients at high risk of SSI with the predictive model and providing prophylactic measures, we could save 398120.7 USD per 1,000 cases of SSI.

**Conclusion:**

We found that ASPi were associated with a reduction in risks of SSI, hospital stays, and cost of antibiotics/treatment in a Vietnamese tertiary hospital.

## 1 Introduction

Surgical site infections (SSI) have always been a concern in many surgeries (1). According to the World Health Organisation (WHO), SSI are one of the most common nosocomial infections, with a prevalence of 0.5–15.0%, depending on the surgery and patient condition (2). In the United States (US), SSI could extend the length of hospitalization by 9.7 days while increasing cost by 20,842 US dollars (USD) per admission, resulting in additional 406,730 hospital-days along with 900 million USD on the national scale (3). Therefore, more resources should be allocated to address this issue.

Given the disease burden of SSI, antimicrobial prophylaxis has been widely applied to prevent morbidity and mortality and reduce the duration and cost of healthcare with minimal adverse drug effects (4). Optimal antimicrobial prophylaxis should be non-toxic, inexpensive, and active against typical pathogens of SSI (4, 5), as well as administered in an appropriate dose and at a proper time to ensure adequate concentration during the surgery (4). However, as reported by some studies, the rates of rational antimicrobial prophylaxis could be less than 20% in some regions (6), possibly because recommendations from clinical guidelines were partially based on weak data or expert opinions (7). This shows a need for a comprehensive approach to manage antibiotic use and other related risks, e.g., SSI and antimicrobial resistance.

Antimicrobial Stewardship Program (ASP), which was called on to implement by the US Centers for Disease Control and Prevention (8), has been repeatedly reported for its superiority in patient outcomes and control of antimicrobial resistance over empirical practice (9–11). While there has been various evidence of ASP’s benefits in high-resource countries worldwide, data from low and middle-income countries have still been limited, particularly in antimicrobial prophylaxis. Therefore, we conducted this study to investigate the real-world effects of ASP interventions (ASPi) in antimicrobial prophylaxis at Nhan Dan Gia Dinh (NDGD) Hospital, a tertiary hospital in Vietnam. This study focused on patients undergoing gastrointestinal surgeries due to the high risk of preventable SSI in this population (12).

## 2 Methods

### 2.1 Study design and data collection

A retrospective cohort study was conducted at 2 surgery facilities (A and B) of the Department of Surgical Gastroenterology (NDGD Hospital), collecting data from July 1st, 2015, to December 31st, 2015. All medical records with the following patient characteristics were collected: (1) aged 18 or above; (2) admission date within the data collection timeframe; and (3) had a clean-contaminated surgery at the Department of Surgical Gastroenterology of the hospital. Records were excluded if the patients: (1) were using treatment antibiotics; (2) were immunocompromised (due to immunosuppressive medications or immunodeficiency disorders); or (3) had signs or symptoms of infections prior to the surgery (evaluated by physicians). We reported this study following the Strengthening the Reporting of Observational Studies in Epidemiology (STROBE) Statement (Supplementary Table S1).

As this was a relatively new practice in Vietnam, the hospital only implemented the ASPi at Facility A as a pilot program while maintaining the standard of care (SoC) at Facility B. Thus, the 2 cohorts in this study were patients treated at Facility B with SoC or at Facility A with SoC + ASPi. All the protocols, guidelines, medical equipment/device, medication supplies, medical support/care, and infection control measures were similar between the 2 facilities. SoC included all relevant medical support and care for patients undergoing gastrointestinal surgeries. Antimicrobial prophylaxis in the SoC cohort was primarily empirical therapy. The SoC + ASPi cohort received guideline-directed or expert-consensus prophylactic antibiotics. Details of the ASPi were presented in Supplementary Table S2.

### 2.2 Outcomes

The primary outcome was 30-day SSI, measured as cumulative incidence. We followed all patients up to 30 days after the surgery for SSI diagnoses that were given by either the surgeons (during hospitalization) or treating physicians (during outpatient visits or rehospitalization). Postoperative mortality within 30 days, unless ruled out by other causes, was also considered an SSI case. The secondary outcomes were length of stay (LoS) after surgery (in days, excluding in-hospital mortality), cost of antibiotics (for both prophylaxis and treatment), and cost of treatment (in USD). We only collected direct medical cost data based on the insurer’s perspective.

### 2.3 Sample size

As the board of directors of our hospital required the full launch of ASPi at all departments in 2016, we had to conduct this study within a short timeframe and limited population size. Thus, we decided to collect all eligible medical records within the last 6 months of 2015. We then performed a power analysis (significance level of 5%) to determine if this sample size were adequate to detect any differences in the outcomes. With 227 patients in the SoC and 168 patients in the SoC + ASPi group, we estimated that the ASPi could reduce the 30-day SSI cumulative incidence from 19.8% (2014 data) to around 10.0%. This resulted in a power of 0.77, which was deemed comparable to the common value of 0.80, considering the short timeframe of this study.

### 2.4 Statistical analysis

All statistical calculations and analyses were performed using R software (version 3.2.3, R Foundation for Statistical Computing, Vienna, Austria). Single imputation was used to address missing data. We presented categorical variables as frequencies with percentages and continuous variables as mean with standard deviation (SD) or median with interquartile range (IQR).

We considered the following covariates as potential confounders: sex (female or male), age (years, <60 or ≥60), body mass index (kg/m^2^), chronic comorbidities (cardiovascular diseases, endocrine diseases, respiratory diseases, gastrointestinal diseases, or cancers), and risks of acquiring multidrug-resistant pathogens (yes or no). The difference in primary outcome was compared using beta-binomial regression, while linear regression was used to analyze the secondary outcomes. Due to the non-normality of LoS after surgery and cost of antibiotics and treatment, we applied bootstrapping with 10,000 replications to estimate the effect differences in these cases. We used Holm method to control for multiple comparisons of outcomes. All statistical tests were performed with a family-wise error rate of 5% and reported with 95% confidence interval (95% CI).

We also built a predictive model to identify patients at high risk for post-discharge SSIs. The variables for modeling include age, gender, days in hospital before/after the surgery, blood transfusion, number and type of comorbidities, serum level of aspartate aminotransferase/alanine aminotransferase/urea/creatinine/glucose, surgery site, surgery type, duration of surgery, American Society of Anesthesiologists (ASA) score, presence of cancer, antimicrobial agent, rational choice/dosage/timing of administration of antibiotics. Four types of models were included and compared, of which the most accurate one would be used for further predictions and estimations. The performances of the models were assessed using: (1) F1 score; (2) accuracy; (3) sensitivity; (4) specificity; (5) positive predictive value (PPV); (6) negative predictive value (NPV) (13). For this predictive modeling, we randomly split the dataset by a ratio of 8:2 into training and testing sets, with the former for model building and the latter for cross-validation. For the data pre-processing, each variable was standardized to a mean of 0 and a variance of 1. Principle component analysis was then used to reduce the dimensionality of the training and testing datasets, of which low-variance (confounding) dimensions were excluded, leaving a total of 95% sum of variances for the remained dimensions. After that, we built and trained our models based on the following methods: (1) logistic regression; (2) random forest; (3) support vector machines (SVM); (4) kernel Fisher discriminant analysis (KFDA) (14). For random forest-based model, we adjusted the cut-off threshold for the labels positive:negative (P:N) by a ratio of 2:8 to prioritize prediction for the P label. With the SVM-based model, we applied the radial basis function kernel, and to avoid overfitting, we set the cost parameter to 10^−6^. Due to the highly unbalanced distribution of P:N labels, instead of using the original F1_1,1_ score, we used the customized F1_1,4_ to improve the P label prediction based on the following equation:


$$F1_{a,b}=\frac{\mathrm{true}\;\mathrm{positive}}{\mathrm{true}\;\mathrm{positive}+\frac{a\times \mathrm{false}\;\mathrm{positive}+b\times \mathrm{false}\;\mathrm{negative}}{a+b}}$$

### 2.5 Ethics approval

This study was approved by the Institutional Review Board (IRB) of NDGD Hospital, under approval number 108/CN-HDDD. The IRB did not require informed consent from patients to conduct this study, as we only used the medical records to collect retrospective data without revealing patient identity.

## 3 Results

### 3.1 Patient and surgery characteristics

**Figure 1:**
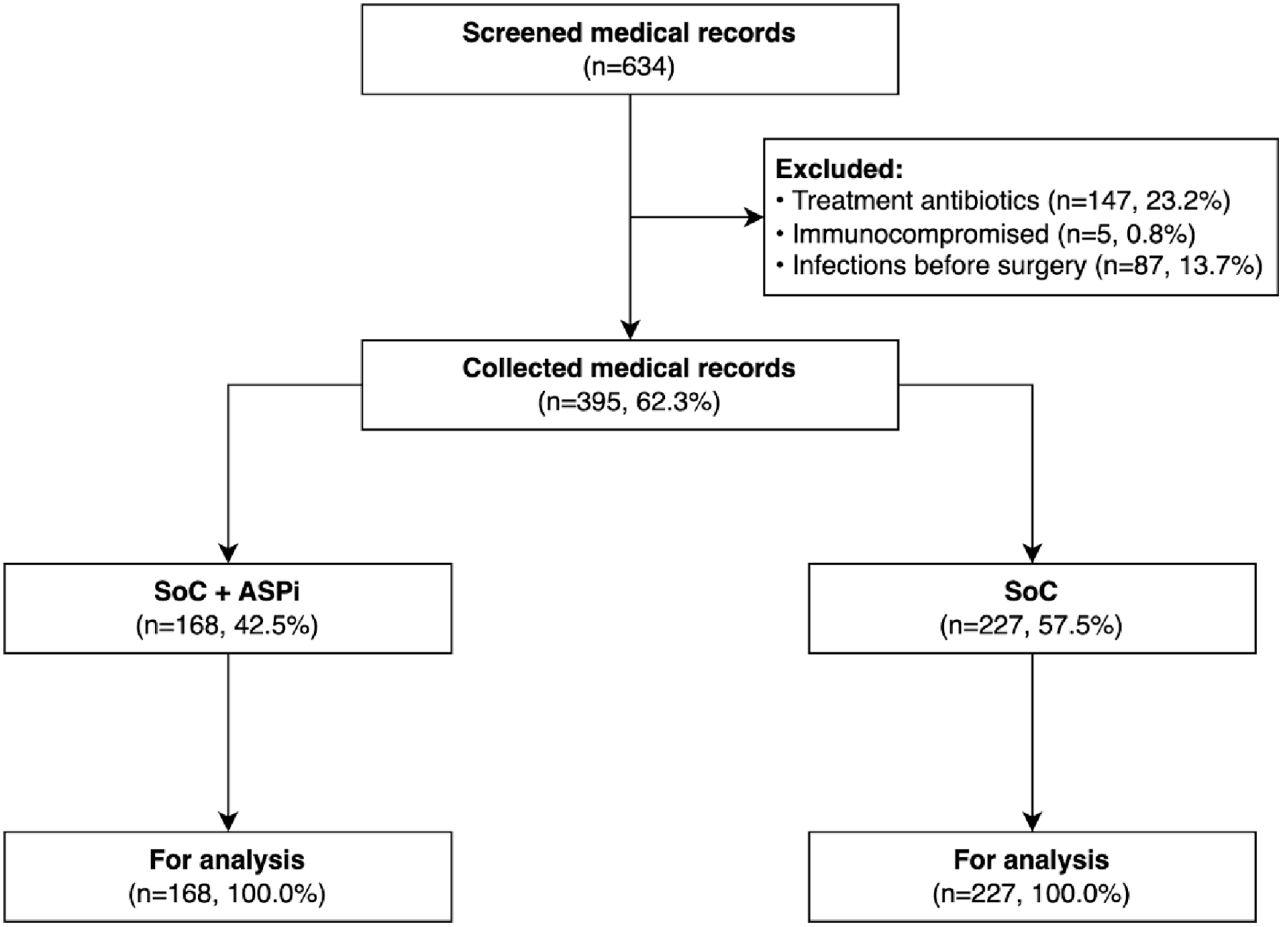
Flowchart of the patients’ medical records. ASPi, interventions of antimicrobial stewardship program; SoC, standard of care.

There were no missing data in this study sample (Figure 1). Overall, 395 patients (48.1% being female) had an average age of 49.4 ± 15.8 years. No patients had an ASA score higher than 3, showing a relatively low risk of SSI in this study setting (15). The majority of patients did not have or have only 1 comorbidity, primarily hypertension (72 out of 395) and diabetes (25 out of 395). Most patients underwent open surgeries (67.1%) in the large intestine (50.4%). Surgery duration varied widely from 15 to 430 min, with a median of 60 min (IQR: 40–100). β-lactam antibiotics were given in most gastrointestinal surgeries, with the predominance of amoxicillin-clavulanic acid in the SoC group and cefazolin in the SoC + ASPi group. Metronidazole was rarely prescribed and was only combined with other antibiotics, including ampicillin/sulbactam, ceftazidime, and ceftriaxone. Among 395 patients, only 3 self-declared to have an antibiotic allergy (2 with penicillin and 1 with cefuroxime). Further details between the SoC and SoC + ASPi groups were reported in Table 1.

**Table 1:** Demographic characteristics of the study sample.

Characteristics	SoC + ASPi (*n* = 168)	SoC (*n* = 227)
**Patient characteristics**		
Age (years), mean ± SD	48.5 ± 15.5	50.1 ± 16.0
**Age category, *n* (%)**		
<60 years	123 (73.2)	158 (69.6)
≥60 years	45 (25.8)	69 (30.4)
**Sex, *n* (%)**		
Female	81 (48.2)	109 (48.0)
Male	87 (51.8)	118 (52.0)
**ASA score, *n* (%)** ^a^^,^ ^b^		
1	68 (40.5)	87 (38.3)
2	81 (48.2)	110 (48.5)
≥3	19 (11.3)	30 (13.2)
**Number of comorbidities, *n* (%)** ^a^		
0	116 (69.1)	148 (65.2)
1	33 (19.6)	46 (20.3)
≥2	19 (11.3)	33 (14.5)
**Surgery characteristics**		
**Surgical site, *n* (%)** ^a^		
Stomach	12 (7.1)	15 (6.6)
Liver–gall bladder–pancreas	21 (12.5)	35 (15.4)
Small intestine	3 (1.8)	2 (0.9)
Large intestine	90 (53.6)	109 (48.0)
Others	42 (25.0)	66 (29.1)
**Types of surgery, *n* (%)**		
Laparoscopic	59 (35.1)	71 (31.3)
Open	109 (64.9)	156 (68.7)
Duration (minutes), median (IQR)	55.0 (33.8–105.0)	60.0 (45.0–112.5)
**Duration category, *n* (%)**		
≤180 min	149 (88.7)	200 (88.1)
>180 min	19 (11.3)	27 (11.9)
**Blood transfusion, *n* (%)**		
Yes	163 (97.0)	218 (96.0)
No	5 (3.0)	9 (4.0)
**Antimicrobial prophylaxis**		
**Penicillin, *n* (%)**		
Amoxicillin-clavulanic acid (875 mg-125 mg, PO)	35 (20.8)	104 (45.8)
Ampicillin-sulbactam (1 g-0.5 g, IV)	9 (5.4)	53 (23.3)
**Cephalosporin, *n* (%)** ^a^		
Cefazolin (2 g, IV)	122 (72.6)	33 (14.5)
Cefoperazone (1 g, IV)	0 (0.0)	1 (0.4)
Ceftazidime (2 g, IV)	1 (0.6)	9 (4.0)
Ceftriaxone (2 g, IV)	0 (0.0)	0 (0.0)
Cefuroxime (1.5 g, IV)	0 (0.0)	21 (9.3)
**Carbapenem, *n* (%)**		
Ertapenem (1 g, IV)	1 (0.6)	4 (1.8)
**Fluoroquinolone, *n* (%)**		
Ciprofloxacin (400 mg, IV or 500 mg, PO)	0 (0.0)	2 (0.9)
**5-nitroimidazole, *n* (%)** ^c^		
Metronidazole (500 mg, IV)	8 (4.8)	2 (0.9)

ASA, American Society of Anesthesiologists; ASPi, interventions of antimicrobial stewardship program; IQR, interquartile range; IV, intravenous route; PO, oral route; SD, standard deviation; SoC, standard of care.

a Percentage may not add up to 100 due to rounding.

b ASA score was calculated using the ASA Physical Status Classification System, available at: https://www.asahq.org/standards-and-guidelines/asa-physical-status-classification-system.

c Metronidazole was only used in combination with other antibiotics and was not counted toward the cumulative percentage.

### 3.2 Primary and secondary outcomes

Table 2 reported the effect estimates for the outcomes of this study. No in-hospital mortality was recorded. We found a significantly lower risk of 30-day SSI (primary outcome) in patients receiving SoC + ASPi (11.9%) than those with SoC (20.7%), with an effect difference of −8.8, 95% CI: −16.0 to −1.6, adjusted *p* = 0.042. We also observed similar results for the secondary outcomes. LoS after surgery was 24.4% shorter following the implementation of ASPi (mean difference of −1.1 days, 95% CI: −1.8 to −0.4, adjusted *p* = 0.004). Healthcare costs were also lower in the SoC + ASPi group (mean difference in cost of antibiotics: −37.3 USD, 95% CI: −59.8 to −14.8, adjusted *p* = 0.012; mean difference in cost of treatment: −191.1 USD, 95% CI: −348.4 to −33.8, adjusted *p* = 0.042).

**Table 2:** Comparison of SSI rate, length of postoperative stay, and cost of antibiotics.

Outcomes	SoC + ASPi	SoC	Estimate (95% CI)^a^
30-day SSI, *n*/total (%)	20/168 (11.9)	47/227 (20.7)	−8.8 (−16.0 to −1.6)^b^
LoS after surgery, days^c^	3.4 ± 2.9	4.5 ± 3.8	−1.1 (−1.8 to −0.4)^d^^,^^e^
Cost of antibiotics, USD^c^	151.4 ± 127.9	188.7 ± 132.4	−37.3 (−59.8 to −14.8)^d^^,^^f^
Cost of treatment, USD^c^	723.8 ± 741.9	914.9 ± 963.2	−191.1 (−348.4 to −33.8)^d^^,^^g^

ASPi, interventions of antimicrobial stewardship program; CI, confidence interval; LoS, length of stay; SoC, standard of care; SSI, surgical site infection; USD, United State dollar.

a All statistical models were controlled for sex, age, body mass index, chronic comorbidities, and risks of acquiring multidrug-resistant pathogens.

b Difference in percentage point was estimated using beta-binomial regression; unadjusted *p* = 0.021; Holm-adjusted *p* = 0.042.

c Presented as mean with standard deviation.

d Estimated using linear regression with bootstrapping (10,000 replications).

e Unadjusted *p* = 0.001; Holm-adjusted *p* = 0.004.

f Unadjusted *p* = 0.004; Holm-adjusted *p* = 0.012.

g Unadjusted *p* = 0.033; Holm-adjusted *p* = 0.042.

### 3.3 SSI predictive modeling

Table 3 summarizes the internal and cross-validated results of SSI predictive modeling by assessing four types of models. Random forest model underwent severe overfitting, as implicated by the significant declines in cross-validity. The KFDA model seemed to outweigh others in terms of F1 score, accuracy, PPV, and NPV. These results suggest the most appropriate model for implementation could be based on the KFDA method.

**Table 3:** Results of SSI predictive models.

	F1 score	ACC	SEN	SPE	PPV	NPV
**Internal validation**						
Logistic regression	58.1	48.0	94.2	39.1	22.9	97.2
Random forest	81.2	81.4	100.0	77.9	46.4	100.0
SVM	54.6	46.4	88.5	38.4	21.6	94.5
KFDA	77.2	84.1	88.1	83.3	51.6	97.2
**Cross-validation**						
Logistic regression	44.4	43.1	72.7	37.7	17.4	88.5
Random forest	47.3	62.5	63.6	62.3	23.3	90.5
SVM	50.5	36.1	90.9	26.2	18.2	94.1
KFDA	69.6	80.1	80.6	80.0	44.9	95.3

ACC: accuracy; KFDA: kernel Fisher discriminant analysis; NPV: negative predictive value; PPV: positive predictive value; SEN: sensitivity; SPE: specificity; SVM: support vector machines.

F1 score was customized to deal with the highly unbalanced distribution of positive over negative labels. All results were reported as percentage (%).

Considering the sensitivity (80.6%) and PPV (44.9%) of the KFDA-based model, we estimated the total reduced cost through the implementation of ASPi and SSI predictive modeling. Patients with a prediction of high risk for SSI would be provided post-discharge prophylaxis (7-day surgical wound care kit, which included sterile gauze pads, antiseptic wipes and swabs, antibiotic ointment, sterile saline solution, medical tape, bandages, surgical gloves, and specialized dressings). In our setting, the expense for this preventive measure was 10.5 USD per patient, while the average cost of SSI treatment was approximately 550 USD per patient. Thus, the implementation of ASPi and SSI predictive modeling could reduce healthcare costs by 398120.7 USD per 1,000 cases of SSI (Supplementary Figure S1).

## 4 Discussion

Following the implementation of ASPi, our study found a significant decrease in all investigated outcomes, i.e., 30-day SSI incidence, LoS after surgery, and cost of antibiotics and treatment. Cross-validated results from the SSI predictive modeling were promising owing to the acceptable sensitivity and PPV. Thus, appropriately targeting patients at high risk for SSI with post-discharge prophylaxis could reduce healthcare costs considerably.

In terms of 30-day SSI incidence, our finding was inconsistent with some previous studies, which observed no remarkable changes in SSI rate before and after implementing the ASPi (16–18). Given the low risk of SSI in these surgeries (19), it might be challenging to detect the true differences (if present) if these studies were not powered to do that. For higher-risk settings, ASPi may be associated with a significant reduction in SSI incidence, as shown in our study or other reports (20, 21). Considering the high rate of SSI after gastrointestinal or abdominal surgeries (12), our results implied that ASPi might be associated with less SSI incidence than empirical practice in patients undergoing high-risk surgeries.

Our finding of shorter LoS after surgery in patients receiving SoC + ASPi was comparable to results of prior reports (11, 21–23). This benefit could be attributable to a lower prevalence of antimicrobial-resistant pathogens (11) or lower SSI incidence (21), assuming that the ASPi were appropriately implemented. Nevertheless, LoS might be mediated by many factors (23), which could cancel out the effects of ASPi. Therefore, even in settings where ASPi were not associated with shorter LoS (24, 25), healthcare institutions should still maintain ASPi standards, unless there is an extremely compelling reason against it.

We found a reduction in cost of antibiotics and treatment after implementing ASPi, which showed consistency with worldwide evidence (11, 18, 23, 25, 26). Given a shorter LoS after surgery and lower SSI incidence, we could totally anticipate the reduced cost of antibiotics in our setting, as reflected in a systematic review (23). Even when SSI incidence did not change significantly, cost of prophylactic antibiotics was still lower in the group with ASPi (18), possibly due to thorough choices of cost-effective medications. Besides, as all patients in our study received the same SoC, the lower cost of treatment was probably a result of lower cost of antibiotics, shorter LoS after surgery, and lower SSI incidence. This saving was critically important for patients in low and middle-income countries.

Given the effectiveness of ASPi at our hospital, we built and cross-validated a predictive model to help identify patients with a high risk of SSIs so that physicians could timely apply appropriate prevention measures. This approach was quite similar to some other prediction models (27–29), which also yielded good estimations. Based on our model’s performance, we estimated our model could further reduce healthcare costs substantially. Despite its potential, due to the skewness of the data, the model encountered an issue of a low PPV. With a fairly high sensitivity to detect most of the high-risk patients, the current proportion of false positives could still be accepted, as the benefits of SSI prevention can outweigh other related risks.

To the best of our knowledge, this study is one of the first to investigate the effects of ASPi in gastrointestinal surgeries in a middle-income Asian country, which could provide more insights into local and regional surgical practice. We controlled for multiplicity in hypothesis testing to generate more robust evidence. Our SSI predictive model also added another layer of benefits for the ASPi by potentially reducing healthcare costs on an institution-wide scale. Despite these findings, our study still has certain limitations. First, indirect costs were not taken into account, which could not comprehensively reflect the economic benefits of ASPi. Second, while physician adherence to guideline-directed therapy is an essential factor of ASPi, this was not covered in our study as the ASPi had not been implemented widely in our hospital at that time. Third, as this study was conducted retrospectively, we could not separate the cognitive biases that might influence the SoC in Facility B. However, as the protocols of ASPi were not widely announced at that time, specifically not Facility B of the Department of Surgical Gastroenterology, we could assume that these biases were not significant. Fourth, cost data were from 2015, which might not best reflect the economical benefits of ASPi. Finally, the performance of our SSI predictive model did not fully meet our expectations, which needed to be improved and externally validated to maximize its combined effectiveness with ASPi.

## 5 Conclusion

We found that appropriately implementing ASPi in gastrointestinal surgeries was associated with a reduction in the risk of SSI, LoS after surgery, and cost of antibiotics and treatment. To increase the impact of ASPi, physician compliance also needs to be promoted and maintained as standard practice. Further studies should explore this aspect for better evidence of ASPi in low-resource settings.

## Data availability statement

The raw data supporting the conclusions of this article will be made available by the authors, without undue reservation.

## Ethics statement

This study was approved by the IRB of Nhan Dan Gia Dinh Hospital. The IRB did not require informed consent from patients to conduct this study, as we only used the medical records to collect retrospective data without revealing patient identity. The study was conducted in accordance with the local legislation and institutional requirements.

## Author contributions

HTP: Conceptualization, Investigation, Methodology, Writing – original draft, Writing – review & editing. T-AM-P: Data curation, Investigation, Writing – original draft, Writing – review & editing, Conceptualization. ADN: Conceptualization, Writing – original draft, Writing – review & editing. V-Q-HN: Data curation, Formal analysis, Writing – original draft, Writing – review & editing. M-HT: Conceptualization, Data curation, Formal analysis, Investigation, Methodology, Supervision, Writing – original draft, Writing – review & editing.
