# Corrigendum: Antimicrobial stewardship program for gastrointestinal surgeries at a Vietnamese tertiary hospital

**DOI:** 10.3389/fmed.2024.1447073

**Published:** 2024-08-12

**Authors:** Hong Tham Pham, Tuong-Anh Mai-Phan, Anh Dung Nguyen, Van-Quang-Huy Nguyen, Minh-Hoang Tran

**Affiliations:** ^1^Faculty of Pharmacy, Nguyen Tat Thanh University, Ho Chi Minh City, Vietnam; ^2^Department of Pharmacy, Nhan Dan Gia Dinh Hospital, Ho Chi Minh City, Vietnam; ^3^Department of Surgical Gastroenterology, Nhan Dan Gia Dinh Hospital, Ho Chi Minh City, Vietnam; ^4^University of Science, VNU-HCM, Ho Chi Minh City, Vietnam; ^5^NTT Hi-Tech Institute, Nguyen Tat Thanh University, Ho Chi Minh City, Vietnam

**Keywords:** antimicrobial stewardship, antimicrobial prophylaxis, gastrointestinal surgery, surgical site infection, Vietnam

In the published article, there was an error in affiliation 1. Instead of “Department of Pharmacy, Nguyen Tat Thanh University, Ho Chi Minh City, Vietnam,” it should be “Faculty of Pharmacy, Nguyen Tat Thanh University, Ho Chi Minh City, Vietnam.”

In the published article, there were errors in the contents of Supplementary Tables S1, S2. For Supplementary Table S1, the locations of the items were not updated in accordance with the final version of the submitted manuscript. For Supplementary Table S2, the information in “Dosage” and “Timing” was incorrect and needed to be modified. The correct Supplementary Tables have been updated in the original article.

In the published article, there was an error. Some variables for modeling have not been mentioned in the previous version. A correction has been made to **Methods**, 2.4 *Statistical analysis*, Paragraph 3. This sentence previously stated:

“The variables for modeling include age, gender, days in hospital before/after the surgery, blood transfusion, number of comorbidities, serum level of aspartate aminotransferase/alanine aminotransferase/urea/creatinine/glucose, surgery site, surgery type, duration of surgery, American Society of Anesthesiologists (ASA) score, presence of cancer, antimicrobial agent, rational choice/dosage/timing of administration of antibiotics.”

The corrected sentence appears below:

“The variables for modeling include age, gender, days in hospital before/after the surgery, blood transfusion, number and type of comorbidities, serum level of aspartate aminotransferase/alanine aminotransferase/urea/ creatinine/glucose, surgery site, surgery type, duration of surgery, American Society of Anesthesiologists (ASA) score, presence of cancer, antimicrobial agent, rational choice/dosage/timing of administration of antibiotics.”

The authors apologize for these errors and state that this does not change the scientific conclusions of the article in any way. The original article has been updated.

