# Supplementary material for: Antimicrobial stewardship program for gastrointestinal surgeries at a Vietnamese tertiary hospital
Source: Front Med (Lausanne). 2024 Apr 17;11:1345698. doi: 10.3389/fmed.2024.1345698 (PMC11061432; doi:10.3389/fmed.2024.1345698)
Supplement: Supplementary file 1 [file Data_Sheet_1.DOCX]

**Antimicrobial stewardship program for gastrointestinal surgeries at a Vietnamese tertiary hospital**

**Authors**

Hong Tham Pham^1,2^, Tuong-Anh Mai-Phan^3^, Anh Dung Nguyen^3^, Van-Quang-Huy Nguyen^4^, Minh-Hoang Tran^5,^*

**Affiliations**

^1^ Faculty of Pharmacy, Nguyen Tat Thanh University, HCMC 72820, Vietnam

^2^ Department of Pharmacy, Nhan Dan Gia Dinh Hospital, HCMC 72316, Vietnam

^3^ Department of Surgical Gastroenterology, Nhan Dan Gia Dinh Hospital, HCMC 72316, Vietnam

^4^ University of Science, VNU-HCM, HCMC 72711, Vietnam

^5^ NTT Hi-Tech Institute, Nguyen Tat Thanh University, HCMC 72820, Vietnam

***Correspondence**

Minh-Hoang Tran

NTT Hi-Tech Institute, Nguyen Tat Thanh University, HCMC 72820, Vietnam

Tel: +84 933342279

Email: tmhoang@ntt.edu.vn


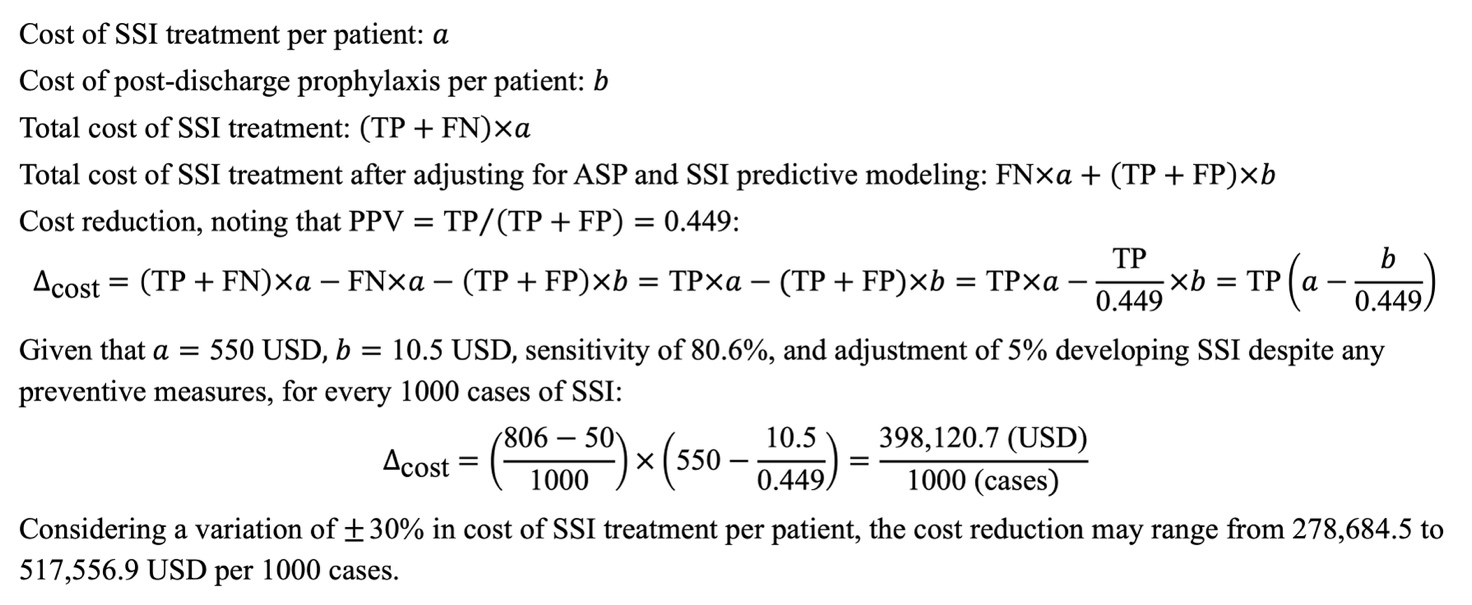


**Figure S1**. Estimation of reduced healthcare cost by implementing ASPi and SSI predictive modeling.

*Abbreviations*: ASPi: interventions of antimicrobial stewardship program; FN: false negative; FP: false positive; PPV: positive predictive value; SSI: surgical site infection; TP: true positive.

*Note*: Cost of SSI treatment and post-discharge prophylaxis per patient were estimated from our clinical data. TP, FP, and FN were taken from the 2x2 table between SSI predictions and observed events. TP+FN refers to observed number of SSI. TP+FP refers to positive predictive number of SSI. Sensitivity is the ratio of TP over TP+FN.

**Table S1.** Checklist of items that should be included in reports of cross-sectional studies.

|  | Item No | Recommendation |
| --- | --- | --- |
| **Title and abstract** | 1 | (*a*) Indicate the study’s design with a commonly used term in the title or the abstract  **Title and abstract** |
|  |  | (*b*) Provide in the abstract an informative and balanced summary of what was done and what was found  **Abstract** |
| Introduction | | |
| Background/rationale | 2 | Explain the scientific background and rationale for the investigation being reported  **Introduction (paragraph 1 and 2)** |
| Objectives | 3 | State specific objectives, including any prespecified hypotheses  **Introduction (paragraph 3)** |
| Methods | | |
| Study design | 4 | Present key elements of study design early in the paper  **Methods (Study design and data collection)** |
| Setting | 5 | Describe the setting, locations, and relevant dates, including periods of recruitment, exposure, follow-up, and data collection  **Methods (Study design and data collection)** |
| Participants | 6 | (*a*) Give the eligibility criteria, and the sources and methods of selection of participants  **Methods (Study design and data collection)** |
| Variables | 7 | Clearly define all outcomes, exposures, predictors, potential confounders, and effect modifiers. Give diagnostic criteria, if applicable  **Methods (Outcomes, Statistical analysis)** |
| Data sources/ measurement | 8* | For each variable of interest, give sources of data and details of methods of assessment (measurement). Describe comparability of assessment methods if there is more than one group  **Methods (Outcomes, Statistical analysis)** |
| Bias | 9 | Describe any efforts to address potential sources of bias  **Methods (Study design and data collection, Outcomes, Statistical analysis)** |
| Study size | 10 | Explain how the study size was arrived at  **Methods (Sample size)** |
| Quantitative variables | 11 | Explain how quantitative variables were handled in the analyses. If applicable, describe which groupings were chosen and why  **Methods (Statistical analysis)** |
| Statistical methods | 12 | (*a*) Describe all statistical methods, including those used to control for confounding  **Methods (Statistical analysis)** |
|  |  | (*b*) Describe any methods used to examine subgroups and interactions  **NA** |
|  |  | (*c*) Explain how missing data were addressed  **Methods (Statistical analysis)** |
|  |  | (*d*) If applicable, describe analytical methods taking account of sampling strategy  **Methods (Sample size)** |
|  |  | (*e*) Describe any sensitivity analyses  **Methods (Statistical analysis)** |
| Results | | |
| Participants | 13* | (a) Report numbers of individuals at each stage of study—eg numbers potentially eligible, examined for eligibility, confirmed eligible, included in the study, completing follow-up, and analysed  **Results (Figure 1)** |
|  |  | (b) Give reasons for non-participation at each stage  **Results (Figure 1)** |
|  |  | (c) Consider use of a flow diagram  **Results (Figure 1)** |
| Descriptive data | 14* | (a) Give characteristics of study participants (eg demographic, clinical, social) and information on exposures and potential confounders  **Results (Table 1, Patient and surgery characteristics)** |
|  |  | (b) Indicate number of participants with missing data for each variable of interest  **Results (Figure 1)** |
| Outcome data | 15* | Report numbers of outcome events or summary measures  **Results (Table 2, Primary and secondary outcomes)** |
| Main results | 16 | (*a*) Give unadjusted estimates and, if applicable, confounder-adjusted estimates and their precision (eg, 95% confidence interval). Make clear which confounders were adjusted for and why they were included  **Results (Table 2)** |
|  |  | (*b*) Report category boundaries when continuous variables were categorized  **NA** |
|  |  | (*c*) If relevant, consider translating estimates of relative risk into absolute risk for a meaningful time period  **NA** |
| Other analyses | 17 | Report other analyses done—eg analyses of subgroups and interactions, and sensitivity analyses  **Results (Table 3)** |
| Discussion | | |
| Key results | 18 | Summarise key results with reference to study objectives  **Discussion (paragraph 1)** |
| Limitations | 19 | Discuss limitations of the study, taking into account sources of potential bias or imprecision. Discuss both direction and magnitude of any potential bias  **Discussion (paragraph 6)** |
| Interpretation | 20 | Give a cautious overall interpretation of results considering objectives, limitations, multiplicity of analyses, results from similar studies, and other relevant evidence  **Discussion (paragraph 2 to 5)** |
| Generalisability | 21 | Discuss the generalisability (external validity) of the study results  **Discussion (paragraph 2 to 5), Conclusion** |
| Other information | | |
| Funding | 22 | Give the source of funding and the role of the funders for the present study and, if applicable, for the original study on which the present article is based  **Funding** |
| *Give information separately for exposed and unexposed groups.  *Note*: An Explanation and Elaboration article discusses each checklist item and gives methodological background and published examples of transparent reporting. The STROBE checklist is best used in conjunction with this article (freely available on the Web sites of PLoS Medicine at http://www.plosmedicine.org/, Annals of Internal Medicine at http://www.annals.org/, and Epidemiology at http://www.epidem.com/). Information on the STROBE Initiative is available at http://www.strobe-statement.org. | | |

**Table S2.** Principles of using prophylactic antibiotics

| **Principle** | **SoC + ASPi** | **SoC** |
| --- | --- | --- |
| Indication | - Clean-contiminated surgeries (procedures involve opening the gastrointestinal, respiratory, or genitourinary or oropharyngeal tract under tightly controlled technical conditions and in the absence of abnormal contamination). - Clean surgeries (surgical procedures involve a normally sterile area of the body; the skin is initially intact; if drainage is required, a closed system must be used; not involve opening of the gastrointestinal, respiratory, genitourinary, or oropharyngeal tract) with elevated risk of SSI. | - Clean-contiminated surgeries (procedures involve opening the gastrointestinal, respiratory, or genitourinary or oropharyngeal tract under tightly controlled technical conditions and in the absence of abnormal contamination). - Clean surgeries (surgical procedures involve a normally sterile area of the body; the skin is initially intact; if drainage is required, a closed system must be used; not involve opening of the gastrointestinal, respiratory, genitourinary, or oropharyngeal tract) with elevated risk of SSI. |
| Choice | - Guideline-directed therapy. - Expert consensus that was based on cumulative antibiogram data. | Empirical therapy. |
| Dosage | - Single dose. - Repeated dose when: (1) surgical duration >twice the half-life of the prophylactic antibiotic; (2) blood loss >1500 mL; or (3) having risk factors of SSI (obesity, diabetes, malnutrition, immunocompromised state, etc.) | - Single dose. - Multiple doses (if elevated risk of SSI after surgery). |
| Route | Intravenous. | Oral or intravenous. |
| Timing | - 30 minutes prior to skin incision. - After surgery if having high risk of SSI. | - 30 minutes prior to skin incision. - After sugery. |
| Abbreviations: ASPi, interventions of antimicrobial stewardship program; SoC, standard of care; SSI, surgical site infection. | | |
